# SARS-CoV-2 and MERS-CoV Spike Protein Binding Studies Support Stable Mimic of Bound 9-*O*-Acetylated Sialic Acids

**DOI:** 10.3390/molecules27165322

**Published:** 2022-08-20

**Authors:** Lisa Oh, Ajit Varki, Xi Chen, Lee-Ping Wang

**Affiliations:** 1 Department of Chemistry, University of California, Davis, CA 95616, USA; 2 Glycobiology Research and Training Center, Departments of Medicine and Cellular and Molecular Medicine, University of California, San Diego, CA 92093, USA

**Keywords:** SARS-CoV-2, MERS-CoV, CoV S protein, sialic acid, MM-PBSA, SOMD, binding free energy simulations, molecular dynamics

## Abstract

Many disease-causing viruses target sialic acids (Sias), a class of nine-carbon sugars known to coat the surface of many cells, including those in the lungs. Human beta coronaviridae, known for causing respiratory tract diseases, often bind Sias, and some preferentially bind to those with 9-*O*-Ac-modification. Currently, co-binding of SARS-CoV-2, a beta coronavirus responsible for the COVID-19 pandemic, to human Sias has been reported and its preference towards α2-3-linked Neu5Ac has been shown. Nevertheless, *O*-acetylated Sias-protein binding studies are difficult to perform, due to the ester lability. We studied the binding free energy differences between Neu5,9Ac2α2-3Galβ*p*NP and its more stable 9-NAc mimic binding to SARS-CoV-2 spike protein using molecular dynamics and alchemical free energy simulations. We identified multiple Sia-binding pockets, including two novel sites, with similar binding affinities to those of MERS-CoV, a known co-binder of sialic acid. In our binding poses, 9-NAc and 9-OAc Sias bind similarly, suggesting an experimentally reasonable mimic to probe viral mechanisms.

## 1. Introduction

Severe acute respiratory syndrome coronavirus 2 (SARS-CoV-2), the beta coronavirus responsible for the COVID-19 pandemic, is structurally highly similar to SARS-CoV-1 (73% sequence identity between spike proteins), yet is remarkably more infective [1]. Both SARS-CoV-1 and SARS-CoV-2 share the same primary human cellular receptor angiotensin-converting enzyme-2 (ACE-2), but this commonality in binding cannot explain the difference in infectivity. One possible mechanism for how SARS-CoV-2 achieves its high infectivity is by binding to sialic acids (Sias), which coat many cell surfaces, especially in the lungs, and are targeted by many disease-causing viruses. In the airways the first encounter of the virus would be with soluble sialomucins, where the very high density of Sias would provide high avidity. On the other hand, the action of cilia are constantly pushing soluble mucins towards the upper airways. Some particles may escape by binding to membrane-bound mucins and sulfated glycosaminoglycans, then are eventually handed off to the high affinity ACE-2 receptors much closer to the plasma membrane.

Sias are part of a large family of over 50 derivatives of the 9-carbon sugar neuraminic acid, where *N*-acetylneuraminic acid (Neu5Ac) is most common [2]. The beta coronavirus Middle East respiratory syndrome coronavirus (MERS-CoV) is known to co-bind to Neu5Ac in addition to its DPP4 primary receptor in a two-step binding mechanism, and depletion of Neu5Ac was found to inhibit MERS-CoV entry into human airway cells [3]. Additionally, MERS-CoV binds preferentially to α2-3-linked Sias over α2-6-linked ones [3]. In another example, beta coronaviruses OC43 and HKUI are known to bind to 9-*O*-acetylated (9-OAc) Sias [1,4,5], where *O*-acetylation is one of the most common Sia modifications found in nature.

While SARS-CoV-1 is not known for its binding to Sias, recent studies have shown Sia binding by the SARS-CoV-2 spike (S) protein, and suggest that sialylated glycans can facilitate viral entry [6,7]. In a lateral flow test, the SARS-CoV-2 S protein bound to both Neu5Ac and α2-3/α2-6-linked Sias, where glycans are each attached to gold nanoparticles (auNP) [8]. While stronger binding under experimental conditions was observed with Neu5Ac-auNP over α2-3/α2-6-linked Sia-auNPs, the report did not preclude involvement of α2-3/α2-6-linked Sias in SARS-CoV-2 S protein binding [8]. Binding studies using sialylated glycans indicate low affinities to the SARS-CoV-2 S protein (~−10 kcal/mol) [9] and specifically along the ACE-2 receptor binding domain (RBD), with mono-sialylated ganglioside glycan affinities of 100–200 μM and multi-sialylated glycan affinities approaching 900 μM, using catch-and-release ESI-MS (CaR-ESI-MS) (~−20 kcal/mol) [6]. A few potential Sia-binding domains have been proposed in addition to the ACE-2 RBD, especially along the flat region of the SARS-CoV-2 S protein *N*-terminal domain (NTD), but specific sites have not been confirmed experimentally [10,11,12,13,14,15,16,17]. The S protein is made up of two subunits: S1 for cell recognition, containing both NTD and ACE-2 RBD, and S2, which is responsible for viral cell membrane fusion [18]. Given the density of Sias along cell surfaces, and the role of S1 in cell recognition, Sias could increase viral binding affinity by acting as an intermediate target or co-binder. We attempt to provide a quick overview of current work on Sias-SARS-CoV-2 S protein binding, where a more thorough study may be found in the recent review by Sun [19].

Experimental studies that test S protein binding to Neu5Ac and *O*-acetylated Sias using glycan microarrays are informative towards understanding the disease and preferential binding [9]. Even so, experimental testing is difficult with *O*-acetylated Sias due to the instability of the ester with respect to migration and cleavage, which depends on pH, temperature, and the presence of esterases [20,21,22,23]. *N*-Acetylated (NAc) Sias have been proposed to be stable synthetic mimics, as they are chemically and structurally similar, as seen in experimental and computational NMR studies [21]. Determining the similarity in S protein binding of 9-NAc to 9-OAc Sias would be valuable when performing binding array studies, and understanding the binding sites of sialic acids to SARS-CoV-2 S protein is important when considering potential druggable sites.

**Figure 1:**
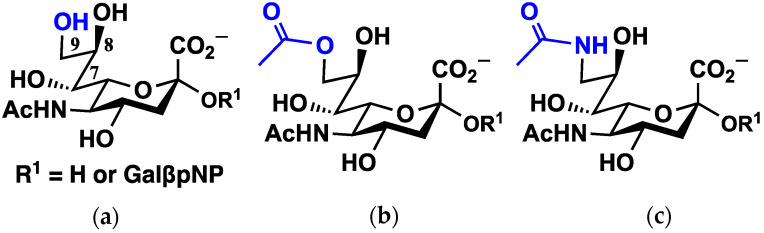
Structures of *N*-acetylneuraminic acid (Neu5Ac) and analogues, the simulated ligands in this study. (**a**) Neu5Ac; (**b**) Neu5,9Ac_2_; and (**c**) Neu5Ac9NAc are simulated ligands, corresponding to the unsubstituted Neu5Ac and its 9-OAc and 9-NAc forms. Two substitutions at R^1^ were simulated, corresponding to the Sia monosaccharide or an α2-3-linked sialoside containing the terminal disaccharide of the GM3 ganglioside, commonly found on cell membranes, followed by *para*-nitrophenol (*p*NP), a molecule used in quantifying Sia binding and cleavage in sialidase activity assays.

**Figure 2:**
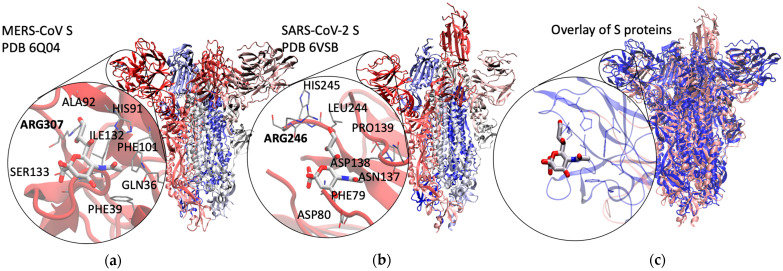
Binding pose in SARS-CoV-2 S protein based on MERS-CoV S protein. (**a**) MERS-CoV S protein with Neu5Ac bound, cryo-EM structure; (**b**) initial binding pose of Neu5Ac in SARS-CoV-2 S protein based on MERS-CoV S protein, used as a starting point for MD simulations; (**c**) structural overlay of pink SARS-CoV-2 S protein (PDB ID: 6VSB) with blue MERS-CoV S protein in complex with Neu5Ac, highlighting binding residues from MERS-CoV S protein (PDB ID: 6Q04).

To this end, we computed the binding free energies of modified Neu5Ac monosaccharides and sialyloligosaccharides to SARS-CoV-2 S protein using molecular dynamics (MD) simulations, starting with a binding pose based on MERS-CoV (Figure 1 and Figure 2). Our simulations revealed new possible Sia-binding sites ranging along the S1 unit, along the NTD and ACE-2 RBD, with some approaching the S2 domain. Each binding site contains a salt bridge connecting a conserved arginine residue to the carboxylate group of Sia, a known motif in Sia–lectin binding [24]; while the binding to individual sites is predicted to be weak, multiple binding to cell surface Sias could strengthen binding overall [1,25]. In addition, we calculated relative binding free energies of ligands that differ in the chemical modification at C9 (9-OH, 9-OAc, and 9-NAc), showing that the synthetic 9-NAc analogues are excellent structural mimics of their naturally occurring 9-OAc counterparts. These insights into Sia-S protein binding could lead to the design of therapeutics that inhibit the binding of S protein to Sias on the cell surface, thereby limiting SARS-CoV-2 transmission.

## 2. Results and Discussion

### 2.1. Method Validation with Sia-MERS-CoV S Protein Binding

Modern free energy simulation methods are estimated to be accurate to within 1–2 kcal/mol for well-behaved [26] protein/ligand systems, but this system presents additional challenges due to the exceptional flexibility of both the SARS-CoV-2 S protein and the ligands, the latter of which we have studied both computationally [20] and using NMR [21]. Based on a recent SAMPL6 challenge that evaluates binding free energy prediction methods [27], we used two methods in this study—an inexpensive implicit solvent approach known as molecular mechanics Poison–Boltzmann surface area (MM-PBSA) [28] to estimate absolute binding free energies, and a more rigorous approach based on alchemical intermediates known as SIRE-OpenMM molecular dynamics (SOMD) [29,30] to compute relative binding free energies of ligands that differ only in the chemical modification at the Sia C9.

**Figure 3:**
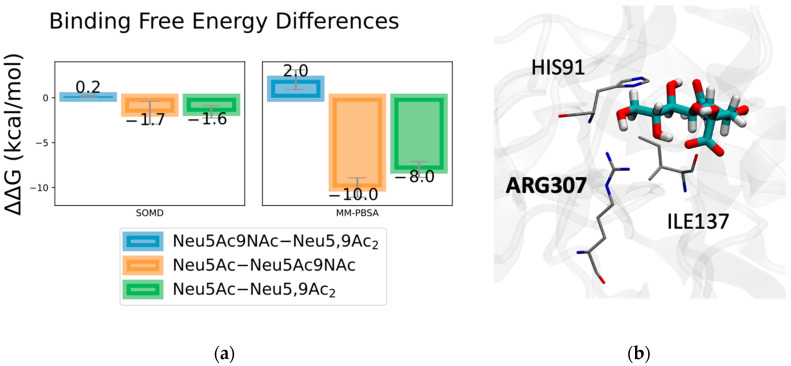
Sia-binding free energy differences in MERS-CoV S protein. (**a**) SOMD and MM-PBSA binding free energy differences for Neu5Ac, Neu5Ac9NAc and Neu5,9Ac_2_ bound in MERS-CoV S protein. Neu5Ac binds stronger than Neu5,9Ac_2_ and Neu5Ac9NAc, where the Neu5Ac9NAc and Neu5,9Ac_2_ result in nominal binding energy differences.; (**b**) Neu5Ac in MERS-CoV S protein binding domain. Top three contributing residues for Neu5Ac are annotated with top contributor bolded. See Figure S3 for energy decomposition results.

Given the exploratory nature of Sia-SARS-CoV-2 S protein binding, we first validated our methods with Sias bound to the RBD in MERS-CoV S protein, known to preferentially bind to Neu5Ac-containing sialosides over Neu5,9Ac_2_- and Neu5Ac9NAc-containing sialosides. For over 1250 ns of combined simulation time, Neu5Ac was observed to stay within the experimentally determined binding site (Neu5Ac remained bound after over 700 ns in a single simulation). Both MM-PBSA binding energy and SOMD alchemical free energy differences show that Neu5Ac binds stronger than either modified variant, consistent with experimental findings (Figure 3). For ~100 ns of combined simulation time, Neu5,9Ac_2_ remained bound, with each simulation unbinding before reaching 40 ns. For ~260 ns of combined simulation time, Neu5,9Ac_2_ remained bound, with over 200 ns in a single simulation. Interestingly, after 70 ns of binding, Neu5Ac9NAc unbound and returned the binding pocket within 16 ns.

In the case of SOMD, Neu5Ac binds stronger than Neu5Ac9NAc and Neu5,9Ac_2_ by 1.6–1.7 kcal/mol. Both Neu5Ac9NAc and Neu5,9Ac_2_ bind very similarly, with a nominal difference of 0.2 kcal/mol, within the margin of error. While the magnitudes of the binding free energy differences are larger in the MM-PBSA results, they follow the same trend as our SOMD results, where Neu5Ac binds stronger than Neu5Ac9NAc and Neu5,9Ac_2_ (by 8–10 kcal/mol), with the modified Sias binding very similarly. Given the shallow nature of the binding pocket, we did not perform binding free energy simulations with the larger sialosides, as the modified Neu5Ac test case is sufficient for validating our methods. As expected from shallow binding and experimental reports for weak binding affinity [31], MM-PBSA binding energy results indicated overall weak binding (−20 kcal/mol for Neu5Ac). Based on energy decomposition analysis, one of the key residues contributing to this difference in binding is ARG307, which binds stronger to Neu5Ac (Figure 3).

Given that the MERS-CoV simulation predictions largely aligned with the experiment, we proceeded toward computational discovery of Sia-binding sites in SARS-CoV-2 S protein and estimation of binding free energies using the same computational approach.

### 2.2. Discovery and Analysis of Sias-SARS-CoV-2 S Protein Binding

**Figure 4:**
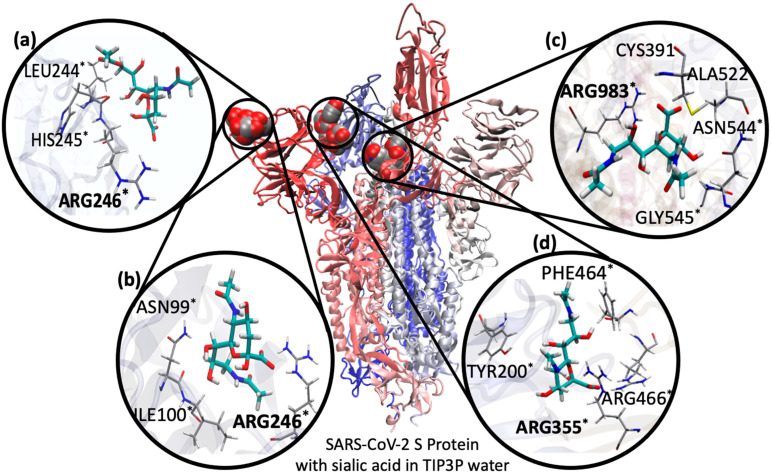
Sia-binding poses with the SARS-CoV-2 S protein. (**a**) Binding pose based on MERS-CoV with Neu5,9Ac_2_ highlighted; (**b**–**d**) binding poses based on initial MD, with Neu5Ac9NAc highlighted. Nearby protein residues are annotated, with top contributor bolded, based on MM-PBSA energy decomposition analysis. * Residues conserved across omicron (PDB ID: 7TB4), kappa (PDB ID: 7VXB), delta (PDB ID: 7W92), gamma (PDB ID: 7M8K), and original (PDB ID: 6VSB) variants of SARS-CoV-2.

We performed MD simulations of Neu5Ac, Neu5,9Ac_2_ and Neu5Ac9NAc bound in SARS-CoV-2 S protein, starting with a docked pose based on the Neu5Ac-MERS-CoV S protein complex (Figure 2, see Methods for details). After initially setting up docked ligands in the cryo-EM structure of SARS-CoV-2 S protein (PDB ID: 6VSB), we found a large RMSD in protein structure after ~200 ns of simulation time indicating a conformational change of the S protein (Figure S1). We observed the single ACE-2 RBD-up moves downwards, and each NTD shrinks inward towards the trimer core, indicating the flexibility of the S1 subunit, specifically the NTD and ACE-2 RBD. Dynamic cross correlation maps support the structural dependence between these regions, specifically between the NTD and ACE-2 RBD-down and NTD and ACE-2 RBD-up regions (Figure S2) [32]. During the final equilibration phase with no restraints, each Sia (Neu5Ac, Neu5,9Ac_2_, and Neu5Ac9NAc) along the NTD of SARS-CoV-2 S protein became unbound. Interestingly, Neu5,9Ac_2_ and Neu5Ac9NAc were observed to return to the original RBD and remained there for ~3 ps before unbinding again. All three Sias subsequently sampled temporary binding events to many regions of the S protein. From the simulation trajectories, we initially selected six poses for further investigation, four of which are in Figure 4.

All of the proposed sites from MD involve arginine forming a salt bridge with the Sia’s carboxylate, a known motif in Sia–lectin binding [24]. In other Sia-S protein observations without an interacting arginine, the Sia quickly dissociated in generally less than 10 ns. While we observed many other binding sites in the MD simulations, such as along the flat top of the NTD within the S1 domain (quick dissociation observed), along the S2 domain and between the S1 and S2 domains (deeper within the S protein trimer), we only considered regions with longer association times and that may be accessible by Sias (see SI video S1 for an overlay of example MD simulations of Sia unbinding and binding events).

Including the initial pose based on MERS-CoV, we chose four binding poses to investigate in closer detail (Figure 4). Here, we describe how each pose was found in our first set of simulations. Structures of Neu5Ac, Neu5,9Ac_2_, Neu5Ac9NAc were docked into the initial binding pocket along the NTD front (based on MERS-CoV, Figure 4a). Given the shallow pocket, each sialic acid unbound and sampled different regions along the S protein. Neu5Ac and Neu5,9Ac_2_ sampled the region behind this initial pose (Figure 4b). This pose is close to pose a, potentially allowing for more accessible Sia-binding domains along the S protein NTD. Neu5Ac9NAc sampled a deeper binding pose between chains A (NTD) and C (ACE-2 RBD) in the initial simulations (Figure 4c). Neu5Ac sampled the region between the ACE-2 RBD and S2 domain and between chains A & B (Figure 4d). This region is accessible for gangliosides to attach to the S protein and direct or inhibit the down to up state of the ACE-2 RBD. Indeed, a previous study indicated that residues along this region may function as a binding pose for glycosaminoglycans including heparan sulfate [33,34,35], highlighting the importance of this region in cellular recognition. Both of these deeper binding domains (Figure 4c,d) overlap with key regions in stabilizing the S protein and affecting its ability to transition the ACE-2 RBD between “down” and “up” states [36]. Sia binding in these regions may shift the S protein flexibility and ability to transition between states, altering ACE-2 binding.

Notably, and to the best of our knowledge, these two Sia-binding poses (Figure 4c,d) have not been previously proposed for Sia binding. One possible explanation is that a number of simulations have been performed using a single NTD or subunit rather than the full S protein trimer, which is needed to describe Sias binding between multiple trimer units (such as between chains A & B in pose d).

**Figure 5:**
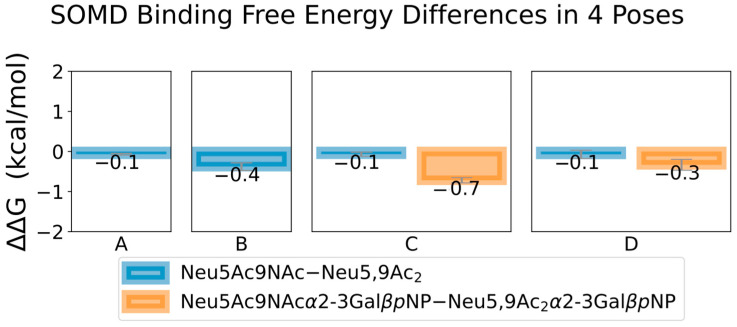
SOMD binding free energy differences for Sias bound in SARS-CoV-2 S protein. Results for transformations between Neu5,9Ac_2_ and Neu5Ac9NAc shown for poses (**A**–**D**), and between Neu5,9Ac_2_α2-3Galβ*p*NP and Neu5Ac9NAcα2-3Galβ*p*NP shown for poses c and d. Error bars are plotted from standard error of means across 4 simulations, when available. See Figure S4 for all SOMD results, including Neu5Ac and Neu5Acα2-3Galβ*p*NP), and Figure S5 for MM-PBSA energies and energy decomposition results.

In all initial MD simulations using the cryo-EM structure, Sia-unbinding events occurred rapidly, given the high flexibility of the SARS-CoV-2 S protein and the resolution of the cryo-EM structure. This flexibility was indicated by relatively fast increase in RMSD at the start of simulations that used the cryo-EM structure. As such, we ran simulations with the SARS-CoV-2 S protein in water to equilibrate the S protein, ideally to a more stable form. We extracted several SARS-CoV-2 S protein structures from the equilibrated trajectories, and used these for docking of the described four poses for production runs, which resulted in longer simulation lengths prior to Sia unbinding. Results from these simulations are in Figure 5.

Figure 5 shows that in each binding pose, Neu5,9Ac_2_ and Neu5Ac9NAc bind very similarly, with differences and errors much less than 1 kcal/mol. Each pose contains a conserved arginine motif, which are conserved across most SARS-CoV-2 variants (omicron (7TB4), kappa (7VXB), delta (7W92), gamma (7M8K), original (6VSB)). Binding residues conserved across these variants are annotated in Figure 4. Neu5Ac binds weaker than either modified Sia in pose a, with slightly weaker binding to LEU244 (Figures S4 and S5 , but each Sia binding strongest to ARG246). In pose b, ARG246 is again the tightest binding residue to the Sias, and Neu5Ac binds more strongly than either Neu5,9Ac_2_ or Neu5Ac9NAc, likely due to its size fitting better in the shallow pocket (Figure S4). In pose C, ARG983 was the tightest binding residue, and ARG355 and ARG466 for pose d. Neu5Ac binds slightly stronger than either modified variant, especially to these key arginine residues (Figure S4). Due to their size, Neu5Acα2-3Galβ*p*NP, Neu5,9Ac_2_α2-3Galβ*p*NP, and Neu5Ac9NAcα2-3Galβ*p*NP in pose c were slightly displaced from the deep ARG983, resulting in the top binding residue as LYS529 (Figure S4). While both Neu5,9Ac_2_ and Neu5Ac9NAc bound weaker than the Neu5Ac, these differences were not large (~1 kcal/mol). In binding pose d, while the Neu5Acα2-3Galβ*p*NP binds stronger than Neu5,9Ac_2_α2-3Galβ*p*NP, the standard error of means is relatively large. Larger error bars calculated for binding poses c and d are due to the range of flexibility observed in the S protein, Sias, and the position and orientation of Sia in the binding pocket.

Based on MM-PBSA binding free energies, Sia binding in each pose of the SARS-CoV-2 S protein is weak (<25 kcal/mol), but is consistent with experimental results [9], and is comparable to that of Sia-MERS-CoV S protein binding (see Figures S4 and S5 for MM-PBSA energies and decomposition analysis). Of all the binding poses, binding pose d resulted in tighter binding of α2-3-linked Sias over their monomer counterparts in its initial equilibrated pose used in SOMD. This highlights the importance of pose d in tighter SARS-CoV-2 S protein co-binding to cell-surface Sias, influencing cellular recognition and ACE-2 binding.

A number of other possible binding sites were observed in the MD simulations, but we did not investigate them further due to the very short dwell times in the trajectories. Weak binding was also observed with ARG158 and ARG237 along the NTD, but the binding pocket was shallow, and dissociation quickly occurred during the equilibration of the Sias-S protein complex. Binding was also observed to ARG328 along the outside of the S below the ACE-2 RBD, but further simulations also showed the site as shallow and a weak binder.

## 3. Methods

### 3.1. Choice of Protein Structures, Sialic Acids, and Binding Poses

The initial protein-ligand complex for molecular dynamics simulations was chosen based on available structures from the Protein Databank. At the start of this study, there were no experimental or computationally modeled SARS-CoV-2 S protein structures with sialic acids bound, to the best of our knowledge. We therefore searched for other human beta coronavirus S protein structures available on the Protein Databank with a bound sialic acid. The MERS-CoV S protein (PDB ID: 6Q04 [3]) has a 35% identity with SARS-CoV-2 S protein, and has an available structure with a bound sialic acid (Neu5Ac). There is no known Sia binding or bound complex with SARS-CoV [1], although it shares a higher sequence identity of 73% with the SARS-CoV-2 S protein (PDB ID: 6VSB [37]).

To generate an initial sialic acid binding pose with the SARS-CoV-2 S protein, we performed sequence alignment with both S proteins (6VSB and 6Q04) using MultiSeq, [38] an extension of the Multiple Alignment tool available in VMD [39], a freely available structural graphics program for visualization and analysis. From the aligned structures and conserved binding residues, notably arginine, we placed the Sia directly into the overlaid SARS-CoV-2 S protein *N*-terminal domain and used the resulting complex as our initial structure (Figure 2).

For additional ligand–protein complexes in both S proteins, we docked Sias (Figure 1) into binding pockets using the OEDocking tool [40,41,42,43]. We docked Neu5Ac, Neu5,9Ac_2_, and Neu5Ac9NAc into each S protein, based on evidence that other beta coronaviruses (OC43 and HKUI [1,4]) prefer to bind Neu5,9Ac_2_ over Neu5Ac, and that *N*-acetylated Sias have shown to be chemically and structurally reasonable mimics to *O*-acetylated counterparts. Given that MERS-CoV S protein preferentially binds to α2-3 over α2-6-linked sialic acids, we docked α2-3-linked sialic acids (Neu5Acα2-3Galβ*p*NP, Neu5,9Ac_2_α2-3Galβ*p*NP, and Neu5Ac9NAcα2-3Galβ*p*NP) into deeper binding pockets of the SARS-CoV-2 S protein, where these new poses were inferred from the molecular dynamics simulations in which a free sialic acid would associate to various residues on the S protein.

### 3.2. Molecular Dynamics Simulation Setup

Molecular dynamics simulations were performed using AMBER18 software suite, using *tleap* for setup and *pmemd.cuda* for dynamics, running on servers equipped with Intel Xeon CPUs and Nvidia GTX 980 Ti or 1080 Ti GPUs [44,45,46,47,48]. We used the ff14SB protein force field, GLYCAM06 carbohydrate force field, the GAFF small molecule force field for portions of the sialosides, and the TIP3P water model. Protein or protein–ligand complexes were solved in a truncated octahedron box with 12.0 Å padding between the biomolecule and simulation cell edge, resulting in around 120,000 water molecules, and Na^+^ ions were added for a net neutral charge. Simulations were run with periodic boundary conditions: a 2.0 fs time step, Langevin thermostat set to 298.15 K, and a collision frequency of 5.0 ps^−1^. The particle mesh Ewald method was used to treat long-range electrostatics with a real-space cutoff of 9.0 Å, and the SHAKE algorithm used to constrain all bonds involving hydrogen. With restraints on complex heavy atoms with a restraint weight of 10 kcal mol^−1^ Å^−2^, 1000 minimization steps were performed, followed by an additional 500 steps with no constraints. We subsequently heated our system from 0.1 K to 298.15 K in a single simulation with equally spaced temperatures across 500 ps, at constant pressure using a Berendsen barostat set to 1.0 atm and a compressibility of 4.5 × 10^−5^ bar^−1^, and harmonic restraints on the protein complex heavy atoms with a force constant of 10 kcal mol^−1^ Å^−2^. Subsequent equilibration was performed at constant pressure using 5 × 100 ns simulations in which the harmonic restraints were set to 10.0, 1.0, 0.1, 0.01, and 0 kcal mol^−1^ Å^−2^, respectively. Three production simulations were performed with the NVT ensemble using the simulation cell volume taken from the final structure of the equilibration run.

Trajectory analyses were carried out using the *cpptraj* [49] simulation analysis package and free energy differences plots generated with *matplotlib* plotting software package in python. Molecular structures were visualized with VMD [39].

The structures used in molecular dynamics simulations were 6VSB and 6Q04, each without sialylation.

### 3.3. Computation of Binding Free Energies Using MM-PBSA and Alchemical Simulation Approaches

This study used a combination of two approaches for computing binding free energies: a relatively approximate and inexpensive Molecular Mechanics Poisson–Boltzmann Surface Area (MM-PBSA) approach to estimate binding free energies and a relatively accurate and computationally expensive approach using alchemical intermediates to estimate binding free energy differences between chemical modifications on the sialic acid ligand.

The MM-PBSA calculations used structures taken from the first set of molecular dynamics simulations, production runs, and the equilibration runs for the alchemical free energy simulations, and results were averaged across all sampled trajectories [50,51,52]. MM-PBSA can be used to calculate free energy differences, where the linearized Poisson–Boltzmann equation is used to compute the solvation free energy components (charge distribution and solvation free energy), and the LCPO method implemented in *sander* is used to generate an empirical term for hydrophobic contributions based on surface area. The binding free energy is the difference between the sum of ligand–protein complex binding and its solvation and the sum of the solvation energies of the free ligand and protein (Figure S6). Implicit solvation in the thermodynamic cycle calculations reduces computational time compared to the explicit water molecules used for solvation in the above alchemical free energy approach. Key interacting protein residues are identified by energy decomposition analysis (EDA). Calculations were performed using MMPBSA.py [30] AMBER software, from molecular dynamics simulation trajectories. Simulations ran for up to 800 ns, and were terminated earlier if the Sia was observed to leave the binding pocket, resulting in an average of 300 ns trajectories for Sias in the SARS-CoV-2 S protein. This was deemed sufficient time for relative binding free energies, as our total simulation lengths sampled in our MM-PBSA calculations are greater than the 1–10 ns range tested as sufficient in a paper studying MM-PBSA protein–ligand binding free energy convergences from crystal structures [50].

### 3.4. Alchemical Free Energy Simulations: Setup and Procedure

Alchemical free energy simulations calculate the free energy difference between two chemical states (i.e., ligand bound vs. unbound) by employing fictitious “alchemical” intermediate states to improve thermodynamic overlap. These simulations were setup in a similar fashion, but employed the automated Sire FESetup [53] tool to prepare systems for alchemical free energy simulations using the Sire and OpenMM software packages (SOMD) [32,54]. Due to differences in the software, there were some differences in the simulation details: we used a rectangular box with a 12.0 Å padding, performed minimization over 300 steps, heated the system from 5.0 K to 298.15 K, and ran equilibration simulations with decreasing constraint force constants in order of 8.0, 6.0, 4.0, 2.0, and 0 kcal mol^−1^ Å^−2^; the first equilibration ran for 100 ps, and subsequent simulations ran for 100 ns, as in the above case. We again used FESetup to generate the simulation inputs for the alchemical intermediates describing the transformation occurring (such as Neu5Ac to Neu5,9Ac_2_). These intermediate systems used dummy atoms and interpolated values of parameters, as well as soft-core potentials to describe partial electrostatic and van der Waals interactions according to the following, Equations (1) and (2) [55]:

r_LJ_ = (2σ_ij_λ + r_ij_^2^)^1/2^, (1)

r_Coul_ = (λ + r_ij_^2^)^1/2^, (2)

Here, r_LJ_ and r_Coul_ are “renormalized” distances that are input into the Lennard-Jones and Coulomb potentials, respectively, ensuring that the potential never reaches the singularity at r = 0, and r_ij_ represents the distance between disappearing/appearing atoms i/j. The value of λ ranges from 0.0 to 1.0, in which λ = 0.0 represents the full interaction with the original ligand’s transforming/disappearing atoms, and λ = 1.0 represents no interaction with these atoms. For the appearing atoms, λ is replaced with 1 − λ, such that interaction is fully turned on at λ = 1.0 (while full interaction is turned off for the disappearing atoms).

Ligands were transformed over 13–21 linearly-spaced λ windows, based on a previous study that used SOMD and other alchemical free energy methods in an assessment of binding affinities [56], and following the scheme outlined in the SI of Loeffler et al. [55] In general, the first set of simulations for each transformation was run with 13 windows, and the second set of simulations with 21 for greater certainty. Each simulation was minimized over 1000 steps prior to 5 ns production run in the NPT ensemble, where free energies were computed from the last 4 ns, based on the assessments from Kuhn et al. and Mey et al. [56,57]. Each transformation was computed in the reverse direction, and simulations were repeated in the forward and reverse directions, starting from the same equilibrated starting complex. For example, Neu5,9Ac_2_ was transformed to Neu5Ac9NAc by slowly turning off the interactions of Neu5,9Ac_2_ and slowly turning them on for Neu5Ac9NAc, over a set of discrete steps, as described the SI of Loeffler et al. [55]. We used *analyse_freenrg* to calculate free energy differences at 298 K using multistate Bennett acceptance ratio (MBAR) and thermodynamic integration (TI) as a reference estimate [58,59,60].

**Figure 6:**
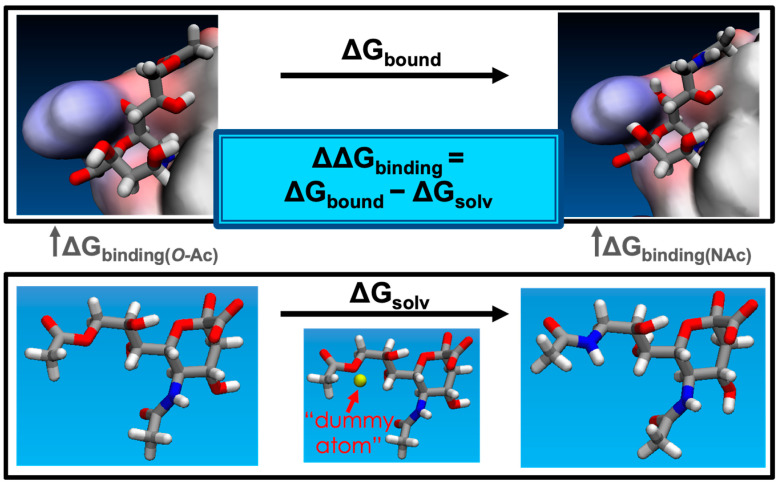
Visual representation of ΔΔG_binding_ of Neu5Ac9NAc versus Neu5,9Ac_2_ in the SARS-CoV-2 S protein, where ΔΔG_binding_ is the difference of two alchemical transformations in explicit solvent (boxed). The upper box is the transformation from Neu5,9Ac_2_ to Neu5Ac9NAc bound in the protein and solvated in explicit water. The lower box is the transformation in explicit water. Additional atoms required in transformations are represented by “dummy atoms” whose interactions are gradually turned on as one progresses over the alchemical intermediates.

The relative free energy of binding is computed as the free energy difference of the alchemical transformation of the ligand bound to the protein and the ligand in solution, i.e., water (Figure 6). The reliability of results was determined based on the similarity between MBAR and TI, and from overlap matrices, where the first off-diagonal element is at least 0.03 [57], but ideally close to 0.10 to ensure enough thermodynamic overlap between simulation windows (Figure S7). Some test cases of longer simulations were performed, but the thermodynamic overlap did not significantly improve compared to more windows. The high degree of flexibility known for the SARS-CoV-2 S protein may contribute to this observation. Thus, we increased the number of windows when necessary, and duplicate simulations were run for higher certainty and increased sampling. Solvated ligand simulations each took about 45 min, and the solvated ligand-protein complex calculations took about 1 day each, running on NVIDIA GTX 980 Ti or 1080 Ti hardware.

### 3.5. Sialic Acid Parametrization Procedure

We parametrized *p*NP with the same approach we used for Neu5,9Ac_2_ and Neu5Ac9NAc in our previous study, which was designed to maximize compatibility with the GLYCAM format [20,61]. Here, we briefly describe the procedure. A set of atomic partial charges was derived for *p*NP. The procedure involves averaging over an ensemble of structures, sampled using a MD simulation of Galβ*p*NP in TIP3P water. We used a GLYCAM06 force field for Gal and GAFF [62] for the para-nitrophenol (*p*NP) aglycon. The system was equilibrated for 1 ns at 298.15 K, 1.0 atm (NPT), followed by a 100 ns production run at 298.15 K (NVT). One hundred structures of Galβ*p*NP were saved at 2 ns intervals and energy-minimized at the HF/6-31G∗ level of theory, with all exocyclic dihedral angles constrained to their MD- sampled values. For each of the 100 constrained energy-minimized structures, electrostatic potential (ESP) calculations were computed using Gaussian16 software [63] and ESP data used in a single-stage restrained ESP fitting (RESP) calculation, with a restraint weight of 0.01 applied to all atoms using the resp program from AmberTools. An arithmetic average over the 100 sets of fitted charges yielded the final set of charges in the model.

The GLYCAM and GAFF force fields lacked torsional parameters to describe the torsional energy profiles about the *p*NP functional group and Galβ–*p*NP linkage; these parameters were derived by fitting to reproduce torsional profiles from DFT at the HF/6- 31G∗ level of theory. We used the TeraChem quantum chemistry software for the energy minimizations and the *torsiondrive* software to scan over the dihedral angles recursively [64,65,66,67,68]. The optimized geometries were used for single-point energies and atomistic forces calculated at the ωB97X-D3/6–31++G(2d,2p) level of theory [69]. The parameters were optimized by fitting to the quantum chemical energies using the ForceBalance optimization software [68,70]. Bond stretching and angle bending parameters for the Galβ*p*NP linkage were copied from analogous parameters available in GLYCAM06. The values of optimized parameters and simulation-ready parameter files are provided in Table S1.

## 4. Conclusions

We have identified multiple weak Sia-binding sites on the SARS-CoV-2 S protein, with two novel Sia-binding poses. Each novel pose, between the NTD and ACE-2 RBD (chains A and C) and between the ACE-2 RBD and S2 domain (chains A and B), is accessible to gangliosides for S protein attachment, and overlaps with regions known in stabilizing the S protein. The binding in each pose is predicted to be weak, in overall agreement with glycan microarray experiments [6,9]. We validated our methods using MERS-CoV-S protein, confirming the experimental result that Neu5Ac binds stronger than that with either 9*-*OAc or 9-NAc modification, and binding energies comparable to Sia-SARS-CoV-2 S protein binding. Each Sia-binding pose in SARS-CoV-2 S protein contains an arginine residue that is conserved across SARS-CoV-2 variants. The multiple Sia-binding sites on SARS-CoV-2 S protein may lead to increased binding affinity to multiple Sias collocated on the cell surface, and the existence of multiple binding sites to the S protein may be validated experimentally. Neu5Ac in SARS-CoV-2 S protein binding sites tend to have stronger binding than their 9-OAc or 9-NAc modifications, but the differences are within the expected margin of error. Binding free energies support 9-NAc Sias as close structural and chemical mimics of 9*-*OAc Sias in SARS-CoV-2 and MERS-CoV S proteins, given the small energy differences (all <1 kcal/mol with SOMD). This supports 9-NAc Sias as an experimentally stable mimic of 9-OAc counterparts to probe Sia-virus binding. Future experimental studies can validate our differential binding free energy results and understanding the binding of modified Sias can further elucidate the role of Sias in cellular recognition and the high transmissibility of SARS-CoV-2.

## Data Availability

The data presented in this study are available in this article.

## Supplementary Materials

The following supporting information can be downloaded at: https://www.mdpi.com/article/10.3390/molecules27165322/s1, Figure S1: Conformational change of the SARS-CoV-2 S protein; Figure S2: Dynamic cross correlation heat map supporting conformational change in the SARS-CoV-2 S protein; Figure S3: MM-PBSA energies and decomposition analysis for Neu5,9Ac2, Neu5Ac9NAc and Neu5Ac in MERS-CoV S protein; Figure S4: SOMD relative binding free energy differences for Sias binding poses with the SARS-CoV-2 S protein; Figure S5: MM-PBSA energies and decomposition analysis for Neu5,9Ac2, Neu5Ac9NAc, Neu5Ac, Neu5,9Ac2α2-3GalβpNP, Neu5Ac9NAcα2-3GalβpNP, and Neu5Acα2-3GalβpNP in all binding poses (a-d) of SARS-CoV-2 S protein; Figure S6: Thermodynamic cycle to estimate binding free energies; Figure S7: Representative binding free energy difference of Neu5Ac—Neu5,9Ac2 in the SARS-CoV-2 S protein; Table S1: Simulation-ready parameter files for the pNP residue in AMBER; Video S1: Overlay of example MD simulations of Sia unbinding and binding events in the SARS-CoV-2 S protein (6VSB).
