# Supplementary material for: SARS-CoV-2 and MERS-CoV Spike Protein Binding Studies Support Stable Mimic of Bound 9-O-Acetylated Sialic Acids
Source: Molecules. 2022 Aug 20;27(16):5322. doi: 10.3390/molecules27165322 (PMC9415320; doi:10.3390/molecules27165322)
Supplement: Supplementary file 1 [file molecules-27-05322-s001.zip › molecules-1866455-supplementary.pdf]

SARS-CoV-2 and MERS-CoV spike protein binding studies support stable mimic of bound 9-*O*-acetylated sialic acids

Lisa Oh,[1] Ajit Varki,[2] Xi Chen,[1] Lee-Ping Wang[1]*

[1] Department of Chemistry, University of California, Davis, California 95616, USA.
[2] Glycobiology Research and Training Center, Departments of Medicine and Cellular and Molecular Medicine, University of California, San Diego, California 92093, USA.

*Address correspondence to: leeping@ucdavis.edu

Table of Contents

Figure S1: *Conformational change of the SARS-CoV-2 S protein* *2*

Figure S2: *Dynamic cross correlation map of SARS-CoV-2 S protein* *2*

Figure S3: *MM-PBSA energies and decomposition analysis for Neu5,9Ac$_2$, Neu5Ac9NAc and Neu5Ac in MERS-CoV S protein* *3*

Figure S4: *SOMD relative binding free energy differences for Sias binding poses with the SARS-CoV-2 S protein* *3*

Figure S5: *MM-PBSA energies and decomposition analysis for Neu5,9Ac$_2$, Neu5Ac9NAc, Neu5Ac, Neu5,9Ac$_2$α2-3GalβpNP, Neu5Ac9NAcα2-3GalβpNP and Neu5Acα2-3GalβpNP in all binding poses (A-D) of SARS-CoV-2 S protein* *4*

Figure S6: *Thermodynamic cycle to estimate binding free energies using MM-PBSA, with Neu5,9Ac$_2$ in protein receptor as example.* *6*

Figure S7: *Representative binding free energy difference of Neu5Ac — Neu5,9Ac$_2$ in the SARS-CoV-2 S protein.* *7*

Table S1: *Simulation-ready parameter files for the pNP residue in AMBER, with torsion parameters fitted to ωB97X-D3/6–31++G(2d,2p) level of theory using the TorsionDrive/ForceBalance software packages (pNP.prep and pNP.frcmod).* *8*

pNP.prep **8**

pNP.frcmod **9**

Figure S1: Conformational change of the SARS-CoV-2 S protein between setup and 200 ns of production simulation time (PDB ID: 6VSB). (a) Initial state of SARS-CoV-2 S protein with a single RBD-up. (b) The structural transformation during 200 ns of simulation, where the opened RBD-up moves downward and NTD's shrink inward towards the trimer core. Structural dependence of these two regions are described in dynamic cross correlation maps in Figure S2.

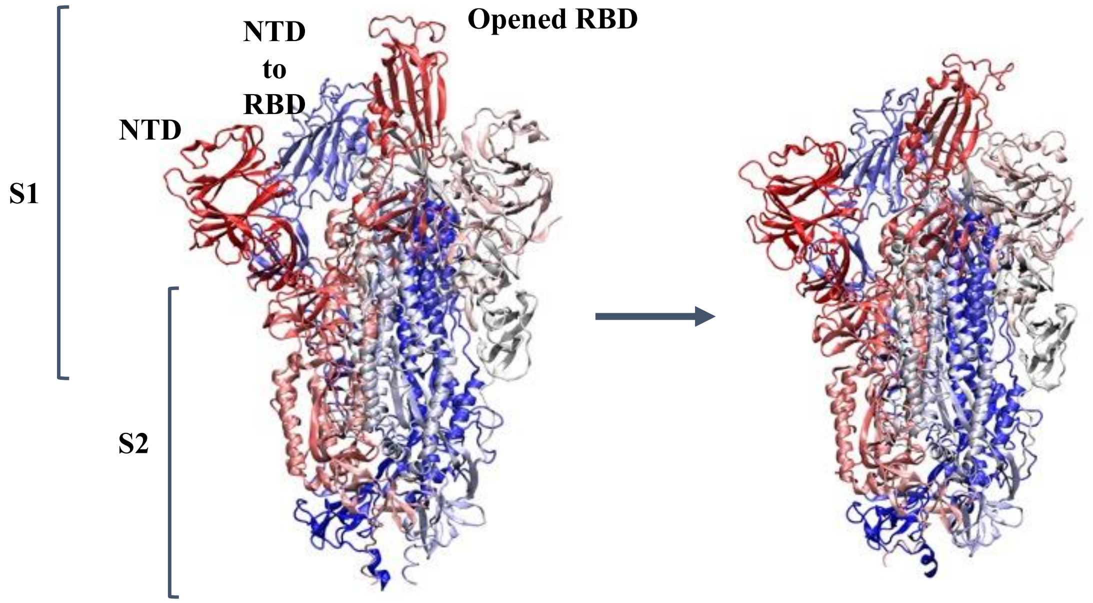

Figure S2: Dynamic cross correlation map of SARS-CoV-2 S protein (PDB ID: 6VSB) in complex with Neu5Ac (not shown), indicating multiple regions of positive correlation throughout 200 ns of initial simulation and using α and β carbons of each residue (See Figure S1). (a) Positive correlation for NTD to ACE-2 RBD-down, regions highlighted in transparent blue (dark and light blue for residues 0 to 200, and 2200 to 2400, respectively); and (b) positive correlation for NTD to ACE-2 RBD-up, regions highlighted in transparent red (dark and light red for residues 240 to 260, and 1000 to 1200, respectively).

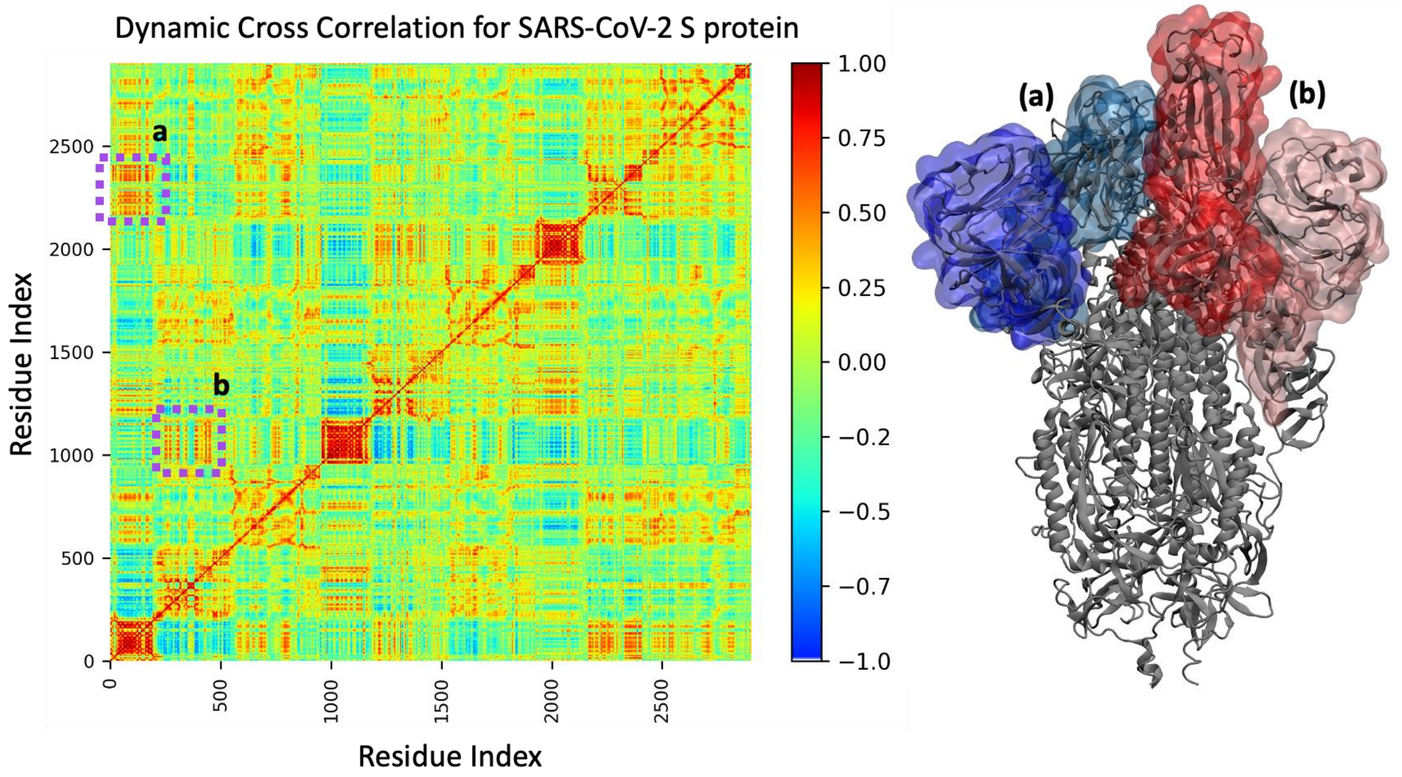

Figure S3: MM-PBSA energies and decomposition analysis for Neu5,9Ac$_2$, Neu5Ac9NAc and Neu5Ac in MERS-CoV S protein, with the top three binding residues are highlighted.

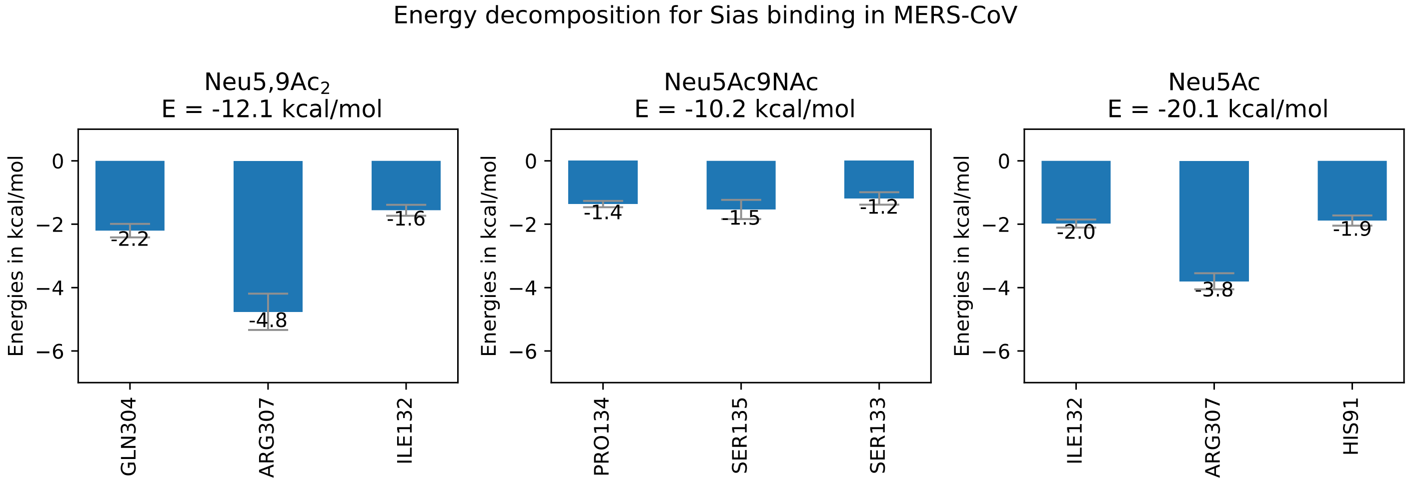

Figure S4: SOMD relative binding free energy differences for Sias binding poses with the SARS-CoV-2 S protein. Results for transformations between Neu5Ac, Neu5,9Ac$_2$, and Neu5Ac9NAc shown for all poses (A-D). Results for transformations between Neu5Acα2-3Galβ*p*NP, Neu5,9Ac$_2$α2-3Galβ*p*NP, and Neu5Ac9NAcα2-3Galβ*p*NP sialosides shown for poses C and D. Error bars are plotted from standard error of means across 4 simulations, when available. Pose D results in stronger binding to Neu5Acα2-3Galβ*p*NP, Neu5,9Ac$_2$α2-3Galβ*p*NP, and Neu5Ac9NAcα2-3Galβ*p*NP over Neu5Ac, Neu5,9Ac$_2$, and Neu5Ac9NAc. See Fig. S3 for MM-PBSA energies and energy decomposition results.

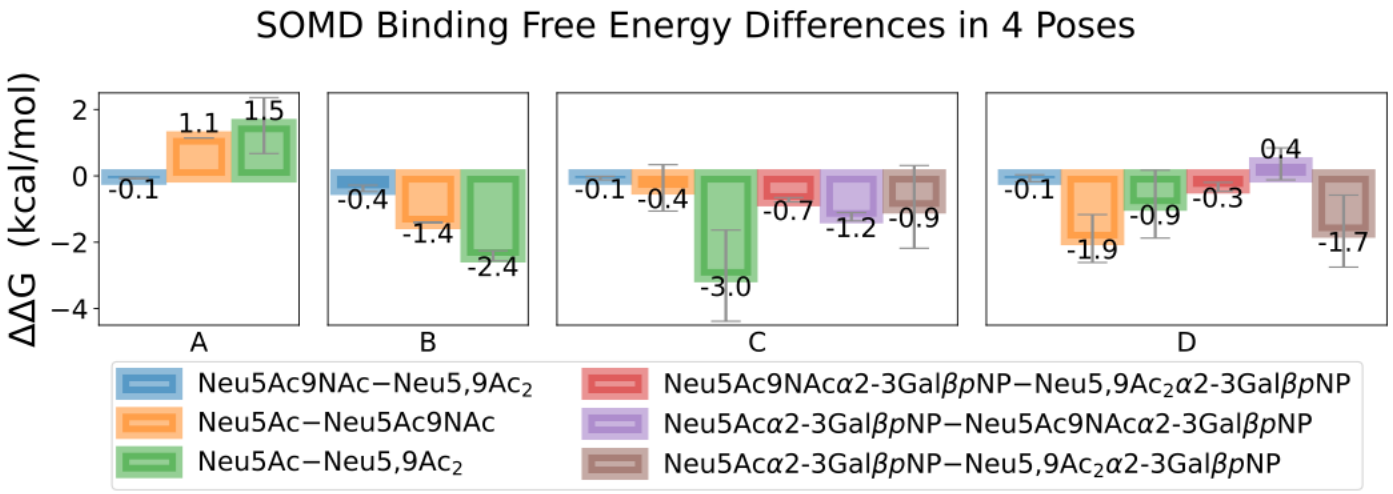

Figure S5: MM-PBSA energies and decomposition analysis for Neu5,9Ac₂, Neu5Ac9NAc, Neu5Ac, Neu5,9Ac₂α2-3Galβ*p*NP, Neu5Ac9NAcα2-3Galβ*p*NP and Neu5Acα2-3Galβ*p*NP in all binding poses (A-D) of SARS-CoV-2 S protein, when available, with the top 3 binding residues are highlighted. Two binding energies are written, one averaged across MD simulations, and the second binding energy from the last equilibration step in the SOMD complex setup is for reference, where the ligands are close to docked locations. The exception is pose B with weak binding and only MM-PBSA energy reported for the SOMD equilibration step. Error bars are plotted from standard error of means.

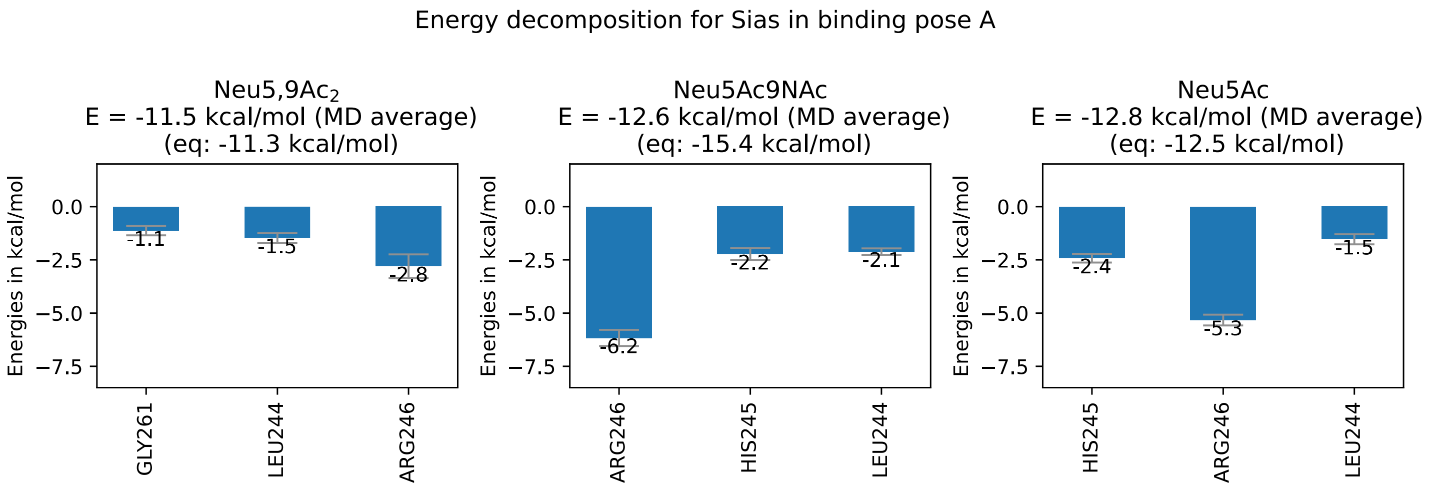

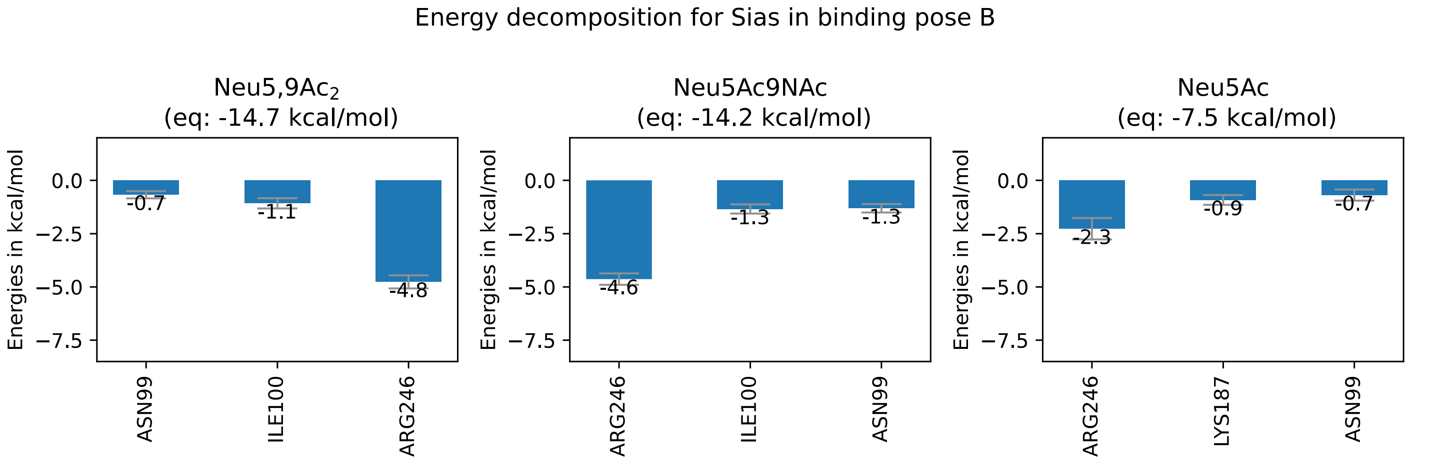

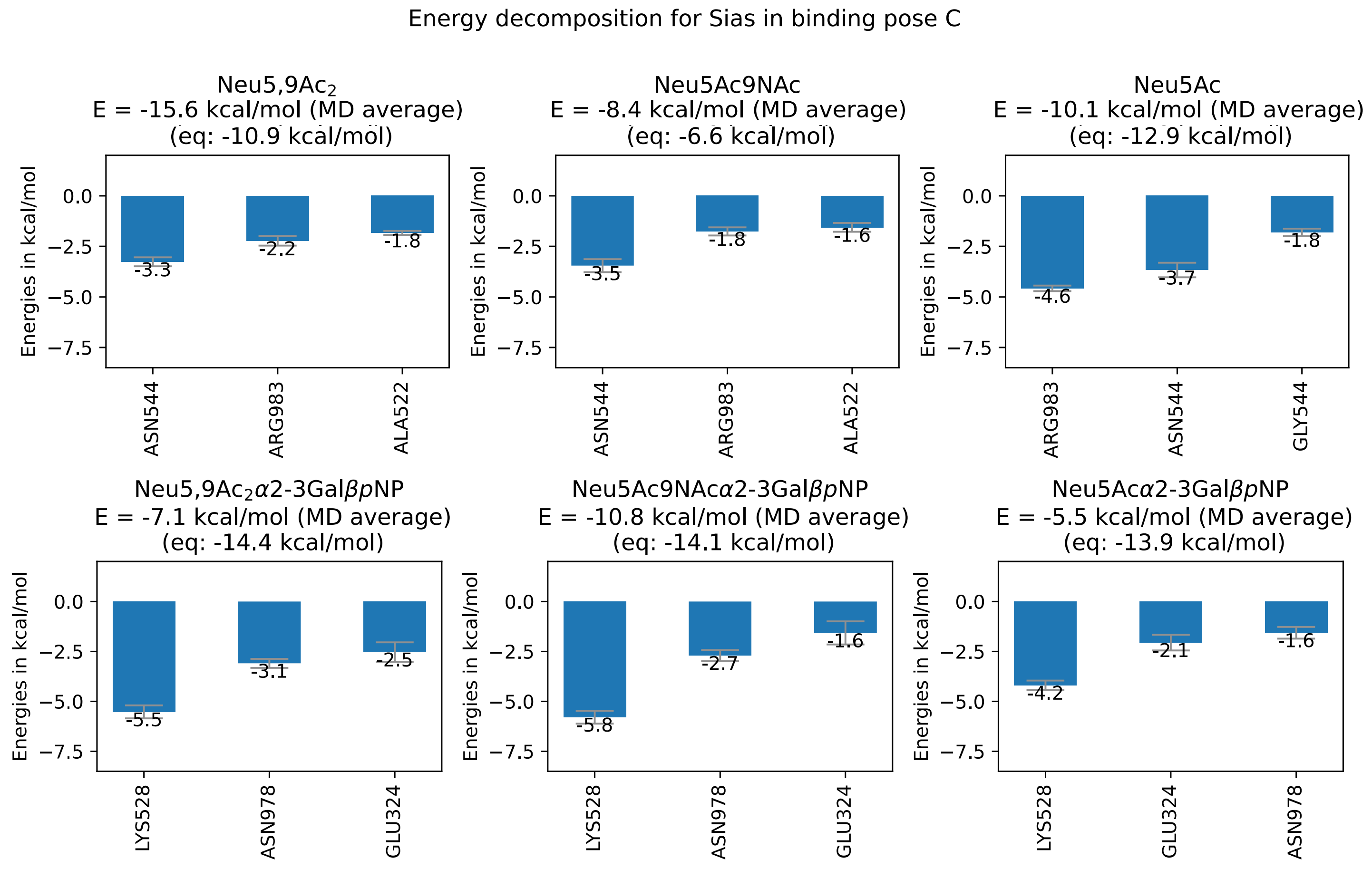
Energy decomposition for Sias in binding pose C
$Neu5,9Ac_2$
E = -15.6 kcal/mol (MD average)
(eq: -10.9 kcal/mol)
Energies in kcal/mol
0.0
−2.5
−5.0
−7.5
-3.3
-2.2
-1.8
ASN544
ARG983
ALA522
Neu5Ac9NAc
E = -8.4 kcal/mol (MD average)
(eq: -6.6 kcal/mol)
-3.5
-1.8
-1.6
ASN544
ARG983
ALA522
Neu5Ac
E = -10.1 kcal/mol (MD average)
(eq: -12.9 kcal/mol)
-4.6
-3.7
-1.8
ARG983
ASN544
GLY544
$Neu5,9Ac_2\alpha 2$-3GalβpNP
E = -7.1 kcal/mol (MD average)
(eq: -14.4 kcal/mol)
-5.5
-3.1
-2.5
LYS528
ASN978
GLU324
Neu5Ac9NAcα2-3GalβpNP
E = -10.8 kcal/mol (MD average)
(eq: -14.1 kcal/mol)
-5.8
-2.7
-1.6
LYS528
ASN978
GLU324
Neu5Acα2-3GalβpNP
E = -5.5 kcal/mol (MD average)
(eq: -13.9 kcal/mol)
-4.2
-2.1
-1.6
LYS528
GLU324
ASN978

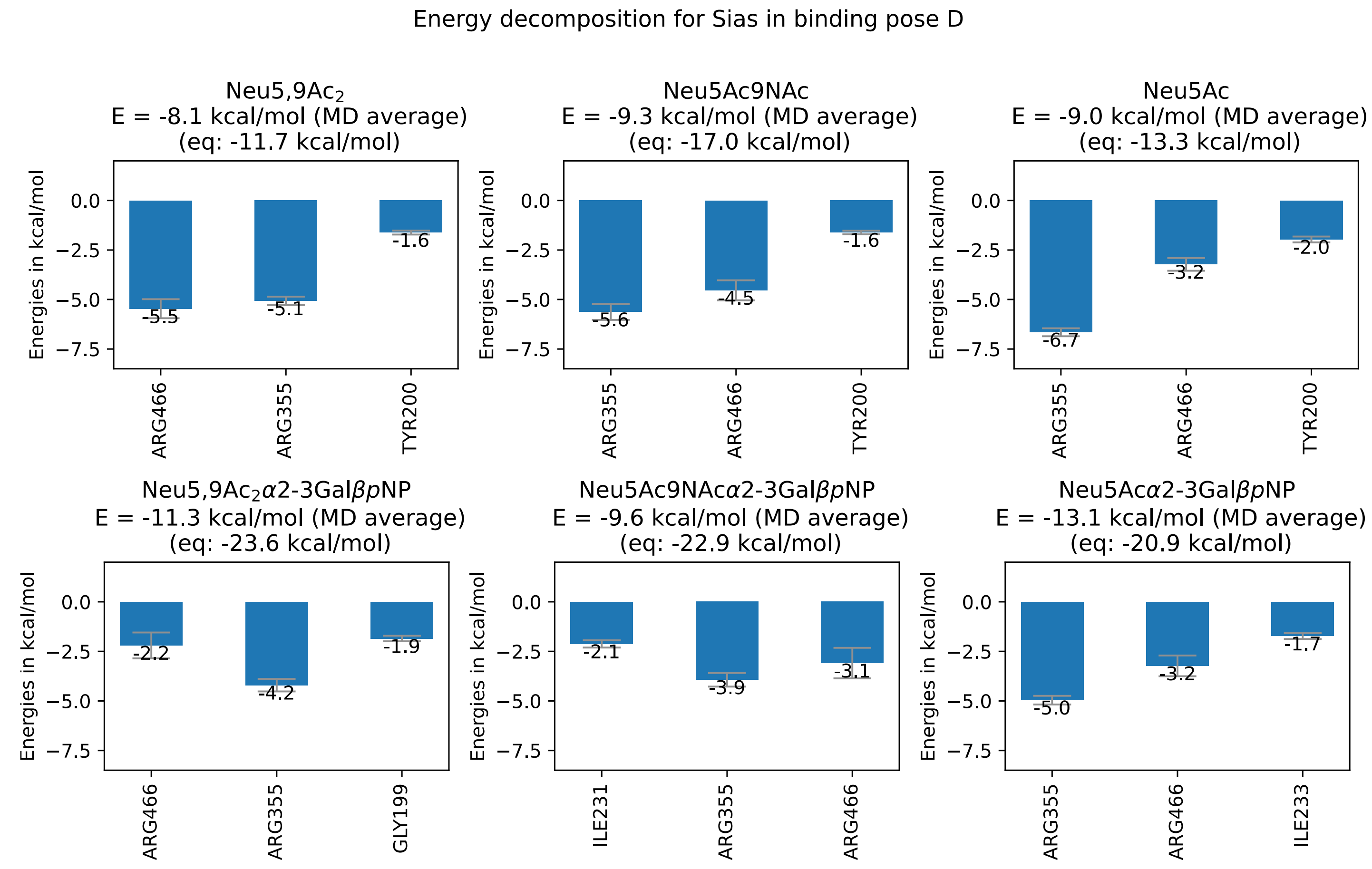
Energy decomposition for Sias in binding pose D
$Neu5,9Ac_2$
E = -8.1 kcal/mol (MD average)
(eq: -11.7 kcal/mol)
Energies in kcal/mol
0.0
−2.5
−5.0
−7.5
-5.5
-5.1
-1.6
ARG466
ARG355
TYR200
Neu5Ac9NAc
E = -9.3 kcal/mol (MD average)
(eq: -17.0 kcal/mol)
-5.6
-4.5
-1.6
ARG355
ARG466
TYR200
Neu5Ac
E = -9.0 kcal/mol (MD average)
(eq: -13.3 kcal/mol)
-6.7
-3.2
-2.0
ARG355
ARG466
TYR200
$Neu5,9Ac_2\alpha 2$-3GalβpNP
E = -11.3 kcal/mol (MD average)
(eq: -23.6 kcal/mol)
-2.2
-4.2
-1.9
ARG466
ARG355
GLY199
Neu5Ac9NAcα2-3GalβpNP
E = -9.6 kcal/mol (MD average)
(eq: -22.9 kcal/mol)
-2.1
-3.9
-3.1
ILE231
ARG355
ARG466
Neu5Acα2-3GalβpNP
E = -13.1 kcal/mol (MD average)
(eq: -20.9 kcal/mol)
-5.0
-3.2
-1.7
ARG355
ARG466
ILE233

Figure S6: Thermodynamic cycle to estimate binding free energies using MM-PBSA, with Neu5,9Ac2 in protein receptor as example.

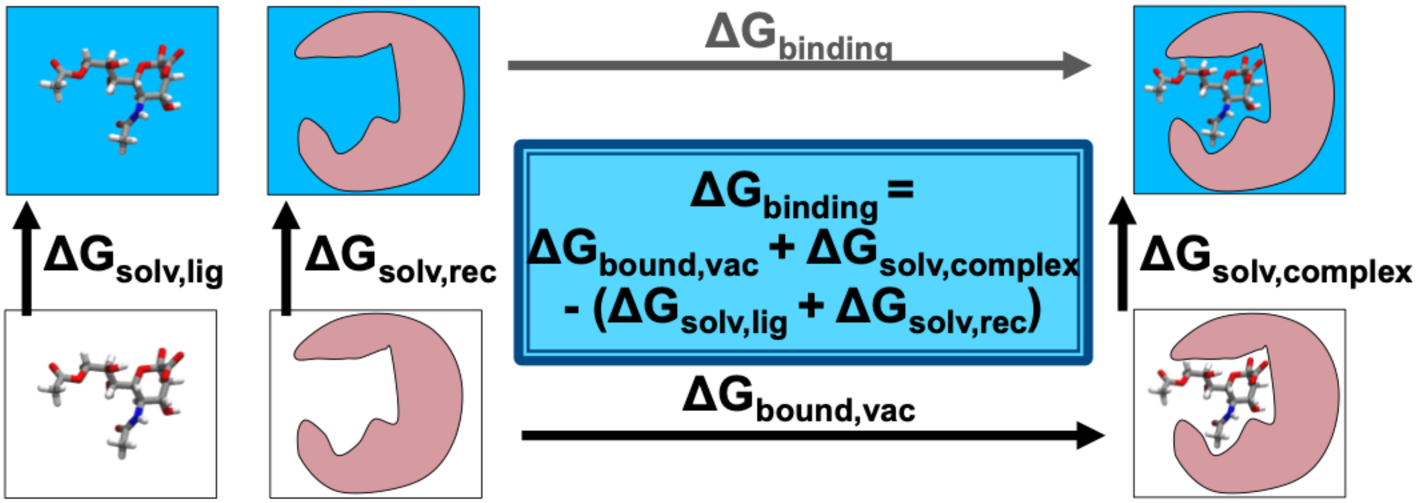

Figure S7: Representative binding free energy difference of Neu5Ac — Neu5,9Ac2 in the SARS-CoV-2 S protein, where the transformation is listed as final – initial Sia. Left side plots display the MBAR and TI energies versus λ. Right side plots display the lower triangles for simulation overlap matrices, where the first off-diagonal quantifies the overlap of one simulation window with the next. Upper plots show analysis for the alchemical transformation of Neu5,9Ac2 to Neu5Ac when bound to the protein and in explicit solvent, and lower plots show results for this transformation in explicit solvent. The binding free energy difference of the Neu5,9Ac2 to Neu5Ac is taken as the difference in energies of the transformations in the bound and solvated systems. Multiple replicates are shown, when available. Plots excluded from analysis when MBAR and TI differ significantly, or in cases where the ligand unbinds during the simulations.

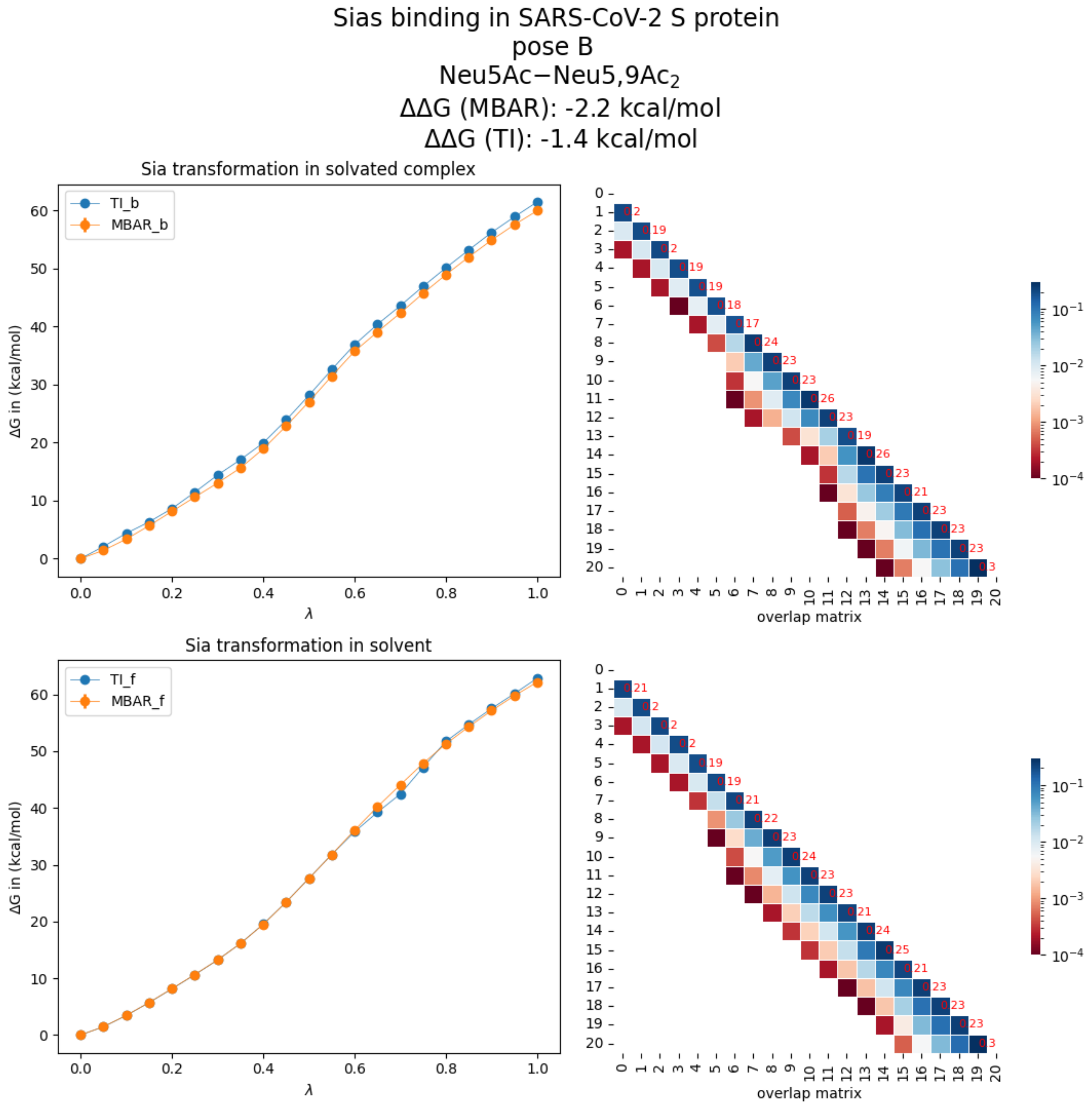

Table S1: Simulation-ready parameter files for the *p*NP residue in AMBER, with torsion parameters fitted to ωB97X-D3/6–31++G(2d,2p) level of theory using the TorsionDrive/ForceBalance software packages (pNP.prep and pNP.frcmod).

pNP.prep

```
   0    0    2

This is a remark line
molecule.res
PNP   INT  0
CORRECT     OMIT DU   BEG
  0.0000
   1  DUMM  DU    M    0  -1  -2     0.000      .0        .0      .00000
   2  DUMM  DU    M    1   0  -1     1.449      .0        .0      .00000
   3  DUMM  DU    M    2   1   0     1.523    111.21      .0      .00000
   4  O1N   o     M    3   2   1     1.540   111.208  -180.000 -0.443190
   5  N     no    M    4   3   2     1.200    43.892    20.983  0.721301
   6  O2N   o     E    5   4   3     1.199   123.189   -42.271 -0.443190
   7  C4    ca    M    5   4   3     1.470   118.375   137.624 -0.013600
   8  C3    ca    B    7   5   4     1.397   119.908    88.427 -0.096035
   9  C2    ca    S    8   7   5     1.394   119.931   179.997 -0.190375
  10  H2    ha    E    9   8   7     1.100   119.977  -179.967  0.151521
  11  H3    ha    E    8   7   5     1.099   120.005    -0.041  0.148947
  12  C5    ca    M    7   5   4     1.395   120.064   -91.611 -0.096035
  13  H5    ha    E   12   7   5     1.099   119.987     0.042  0.148947
  14  C6    ca    M   12   7   5     1.395   119.983  -179.952 -0.190375
  15  H6    ha    E   14  12   7     1.099   119.989   179.945  0.151521
  16  C1    ca    M   14  12   7     1.395   120.031     0.000  0.278468
  17  O     os    M   16  14  12     1.430   120.023  -179.980 -0.321903


LOOP
   C1   C2

IMPROPER
   C4   O1N   N  O2N
   C5   C3   C4    N
   C4   C2   C3   H3
   C1   C3   C2   H2
   C6   C4   C5   H5
   C5   C1   C6   H6
   C6   C2   C1    O

DONE
STOP
```

pNP.frcmod

```
Remark line goes here
MASS
os 16.000       0.465
ca 12.010       0.360
ha 1.008        0.135
no 14.010       0.530
o  16.000       0.434

BOND
ca-os  376.60   1.370
ca-ca  461.10   1.398
ca-ha  345.80   1.086
ca-no  321.70   1.469
no-o   741.80   1.226
Cg-os  266.9    1.442         # PRM 1 2

ANGLE
ca-ca-os   69.600     119.200
ca-ca-ca   66.600     120.020
ca-ca-ha   48.200     119.880
ca-ca-no   66.800     119.010
ca-no-o    68.700     117.760
o -no-o    76.700     125.080
H2-Cg-os    59.50     109.62       # PRM 1 2
os-Cg-Os    99.34     111.22       # PRM 1 2
os-Cg-Cg    69.75     112.51       # PRM 1 2
Cg-os-ca    57.9      121.69       # PRM 1 2

DIHE
ca-ca-ca-os     4   14.500      180.000          2.000
ha-ca-ca-os     4   14.500      180.000          2.000
ca-ca-ca-ca     4   14.500      180.000          2.000
ca-ca-ca-ha     4   14.500      180.000          2.000
ca-ca-ca-no     4   14.500      180.000          2.000
ca-ca-no-o      4   -0.038      180.000          -4.000      # PRM 2
ca-ca-no-o      4   2.391       180.000          2.000       # PRM 2
ha-ca-ca-ha     4   14.500      180.000          2.000
ha-ca-ca-no     4   14.500      180.000          2.000
os-Cg-Os-Cg   1   0.96        0.0              -3.       SCEE=1.0 SCNB=1.0 From GLYCAM Os-Cg-Os-Cg
              1   1.38        0.0              -2.       SCEE=1.0 SCNB=1.0
              1   1.08        0.0              1.        SCEE=1.0 SCNB=1.0
os-Cg-Cg-Cg   1   -0.27       0.0              1.        SCEE=1.0 SCNB=1.0 From GLYCAM Os-Cg-Cg-Cg
H1-Cg-Cg-os   1   0.05        0.0              3.        SCEE=1.0 SCNB=1.0 From GLYCAM H1-Cg-Cg-Os
Oh-Cg-Cg-os   1   -1.10       0.0              -1.       SCEE=1.0 SCNB=1.0 From GLYCAM Oh-Cg-Cg-os
              1   0.25        0.0              2.        SCEE=1.0 SCNB=1.0
H2-Cg-os-ca   3   4.114       -3.772           3.000      # PRM 2 3
Os-Cg-os-ca   3   -0.088      0.150            3.000      # PRM 2 3
Cg-Cg-os-ca   3   -2.196      1.482            -3.000     # PRM 2 3
Cg-Cg-os-ca   3   -2.264      -24.973          -2.000     # PRM 2 3
Cg-Cg-os-ca   3   4.775       -20.104          1.000      # PRM 2 3
```

ca-ca-os-Cg	2	0.029	180.000	-4.000	# PRM 2
ca-ca-os-Cg	2	2.798	180.000	2.000	# PRM 2

IMPROPER

ca-ca-ca-os	1.1	180.0	2.0	Using the default value
ca-ca-ca-ha	1.1	180.0	2.0	Using general improper torsional angle X- X-ca-ha, penalty score= 6.0)
ca-ca-ca-no	1.1	180.0	2.0	Using the default value
ca-o -no-o	1.1	180.0	2.0	Using the default value

NONBON

os	1.6837	0.1700
ca	1.9080	0.0860
ha	1.4590	0.0150
no	1.8240	0.1700
o	1.6612	0.2100
